# Pore-scale Imaging and Characterization of Hydrocarbon Reservoir Rock Wettability at Subsurface Conditions Using X-ray Microtomography

**DOI:** 10.3791/57915

**Published:** 2018-10-21

**Authors:** Amer M. Alhammadi, Ahmed AlRatrout, Branko Bijeljic, Martin J. Blunt

**Affiliations:** ^1^Department of Earth Science and Engineering, Imperial College London

**Keywords:** Engineering, Issue 140, Wettability, contact angle, X-ray microtomography, pore scale, multiphase flow, subsurface conditions, segmentation.

## Abstract

*In situ* wettability measurements in hydrocarbon reservoir rocks have only been possible recently. The purpose of this work is to present a protocol to characterize the complex wetting conditions of hydrocarbon reservoir rock using pore-scale three-dimensional X-ray imaging at subsurface conditions. In this work, heterogeneous carbonate reservoir rocks, extracted from a very large producing oil field, have been used to demonstrate the protocol. The rocks are saturated with brine and oil and aged over three weeks at subsurface conditions to replicate the wettability conditions that typically exist in hydrocarbon reservoirs (known as mixed-wettability). After the brine injection, high-resolution three-dimensional images (2 µm/voxel) are acquired and then processed and segmented. To calculate the distribution of the contact angle, which defines the wettability, the following steps are performed. First, fluid-fluid and fluid-rock surfaces are meshed. The surfaces are smoothed to remove voxel artefacts, and *in situ* contact angles are measured at the three-phase contact line throughout the whole image. The main advantage of this method is its ability to characterize *in situ* wettability accounting for pore-scale rock properties, such as rock surface roughness, rock chemical composition, and pore size. The *in situ* wettability is determined rapidly at hundreds of thousands of points.

The method is limited by the segmentation accuracy and X-ray image resolution. This protocol could be used to characterize the wettability of other complex rocks saturated with different fluids and at different conditions for a variety of applications. For example, it could help in determining the optimal wettability that could yield an extra oil recovery (*i.e.*, designing brine salinity accordingly to obtain higher oil recovery) and to find the most efficient wetting conditions to trap more CO_2_ in subsurface formations.

**Figure:**
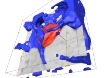


## Introduction

Wettability (the contact angle between immiscible fluids at a solid surface) is one of the key properties that control fluid configurations and oil recovery in reservoir rocks. Wettability affects macroscopic flow properties including relative permeability and capillary pressure^1^,^2^,^3^,^4^,^5^,^6^. However, measuring the *in situ* wettability of reservoir rock has remained a challenge. Reservoir rock wettability has been determined traditionally at the core scale, indirectly using wettability indices^7^,^8^, and directly *ex situ* on flat mineral surfaces^4^,^9^,^10^,^11^. Both wettability indices and *ex situ* contact angle measurements are limited and cannot characterize the mixed-wettability (or range of contact angle) that typically exist in hydrocarbon reservoirs. Moreover, they do not account for pore-scale rock properties, such as rock mineralogy, surface roughness, pore-geometry, and spatial heterogeneity, that have a direct impact on the fluid arrangement at the pore scale.

Recent advances in non-invasive three-dimensional imaging using X-ray microtomography^12^, in combination with the use of an elevated temperature and pressure apparatus^13^, have allowed the study of multiphase flow in permeable media^14^,^15^,^16^,^17^,^18^,^19^,^20^,^21^,^22^,^23^. This technology has facilitated the development of manual *in situ* contact angle measurements at the pore scale in an opaque porous medium (quarry limestone rock) at subsurface conditions^24^. A mean contact angle value of 45° ± 6° between CO_2_ and potassium iodide (KI) brine was obtained by hand from raw images at 300 points. However, the manual method is time-consuming (*i.e.*, 100 contact angle points could take up to several days to be measured) and the values obtained could have a subjective bias.

The measurement of an *in situ* contact angle has been automated by different methods applied to segmented three-dimensional X-ray images^25^,^26^,^27^. Scanziani* et al.*^25^ improved the manual method by placing a circle at the fluid-fluid interface that intersects with a line placed at the fluid-rock interface on slices orthogonal to the three-phase contact line. This method has been applied to small sub-volumes extracted from three-dimensional images of quarry limestone rock saturated with decane and KI brine. Klise* et al.*^26^ developed a method to quantify the *in situ* contact angle automatically by fitting planes to the fluid-fluid interfaces and fluid-rock interfaces. The contact angle was determined between these planes. This method was applied to three-dimensional images of beads saturated with kerosene and brine. Both automated methods were applied to voxelized images that might introduce error, and in both methods, lines or planes were fitted at the fluid-fluid and fluid-rock interfaces and the contact angle was measured between them. Applying these two approaches on voxelized segmented images of complex rock geometry could lead to errors while also being time-consuming.

In this protocol, we apply the automated *in situ* contact angle method developed by AlRatrout* et al.*^27^ that removes voxelization artefacts by applying Gaussian smoothing to the fluid-fluid and fluid-solid interfaces. Then, a uniform curvature smoothing is applied only to the fluid-fluid interface, which is consistent with the capillary equilibrium. Hundreds of thousands of contact angle points are measured rapidly in combination with their *x*-, *y*-, and *z*-coordinates. The approach of AlRatrout* et al.*^27^ has been applied to water-wet and mixed-wet quarry limestone samples saturated with decane and KI brine.

In this protocol, we employ the latest advances in X-ray microtomography combined with a high-pressure and high-temperature apparatus to conduct an *in situ* wettability characterization of complex carbonate reservoir rocks, extracted from a very large producing oil field located in the Middle East. The rocks were saturated with crude oil at subsurface conditions to reproduce the reservoir conditions upon discovery. It has been hypothesized that parts of the reservoir rock surfaces (with direct contact with crude oil) become oil-wet, while others (filled with initial formation brine) remain water-wet^28^,^29^,^30^. However, the reservoir rock wettability is even more complex due to several factors controlling the degree of wettability alteration, including the surface roughness, the rock chemical heterogeneity, the crude oil composition, the brine composition and saturation, and the temperature and pressure. A recent study^31^ has shown that there is typically a range of contact angle in reservoir rocks with values both above and below 90°, measured using the automated method developed by AlRatrout* et al.*^27^.

The main objective of this work is to provide a thorough protocol to characterize the *in situ* wettability of reservoir rocks (mixed-wettability) at subsurface conditions. An accurate measurement of an *in situ* contact angle requires a good segmentation quality. Hence, a machine learning-based segmentation method known as Trainable WEKA Segmentation (TWS)^32^ was used to capture not only the amount of remaining oil but also the shape of the remaining oil ganglia, thus facilitating more accurate contact angle measurements. Recently, TWS has been used in a variety of applications, such as the segmentation of packed particle beds, liquids within textile fibers, and pores of tight reservoirs^33^,^34^,^35^,^36^,^37^,^38^,^39^,^40^. To image the remaining oil accurately at a high resolution and at subsurface conditions, a novel experimental apparatus was used (Figure 1 and Figure 2). Mini-samples of rock were loaded into the center of a Hassler-type core holder^41^ made of carbon fiber. The use of a long and small diameter carbon fiber sleeve allows an X-ray source to be brought very close to the sample, hence increasing the X-ray flux and reducing the required exposure time, resulting in a better image quality in a shorter period of time. The carbon fiber sleeve is strong enough to handle high pressure and temperature conditions while remaining sufficiently transparent to X-rays^21^.

In this study, we outline the steps followed to characterize the *in situ* wettability of reservoir rocks at subsurface conditions. This includes drilling representative mini-samples, the core holder assembly, the flow apparatus and flow procedure, the imaging protocol, the image processing and segmentation, and finally running the automated contact angle code to generate contact angle distributions.

## Protocol

### 1. Drilling Representative Mini-samples of Rock

1. To acquire high-resolution scans, drill mini-samples (*i.e.*, with a diameter of 5 mm and a length of 15 - 30 mm). Firstly, label the core plug with 2 reference marks orthogonal to each other as shown in Figure 3. Then, acquire a full field-of-view (FFOV) scan of the core plug with a voxel size of 40 µm/voxel to visualize the internal distribution of pores and grains.
2. Identify and label good drilling locations carefully: these avoid large vugs or mineral grains. Use a data visualization and analysis software (**Table of Materials**) to visualize the three-dimensional image of the rock as shown in Figure 3. Open a two-dimensional slice of the rock dry image and identify good drilling locations while moving the slice from the top to the base of the rock.
3. Use a stainless-steel drilling bit to drill the mini-samples while using running water as a cooling fluid. Extract the fragile mini-samples carefully, using a thin chisel (*i.e.*, a small flat head screwdriver) to remove the mini-samples from their base. Make both ends of the mini-samples flat to facilitate good contact with the flow end pieces.
4. Measure the dimensions of the mini-samples accurately using a caliper. Use the measured dimensions to calculate the bulk volume. Multiply the measured bulk volume by the measured helium porosity to find the pore volume.
5. To measure the helium porosity of the mini-samples, use a gas pycnometer. First, use the gas pycnometer to measure the grain density (kg/m^3^) of the dry rock sample. Divide the mass (kg) of the dry sample by the measured grain density (kg/m^3^) to obtain the grain volume (m^3^). Subtract the grain volume from the bulk volume calculated in step 1.4 and, finally, divide the difference by the bulk volume to obtain the total porosity (fraction).
6. Scan the drilled mini-samples at a higher resolution (*i.e.*, 5.5 µm/voxel) using an X-ray microtomography scanner to assess the internal pore structure. Refer to step 4 for more details on how this is done. NOTE: Drilling mini-samples involves moving mechanical parts. So, wear complete personal protective equipment (PPE) and take appropriate precautions while drilling.

### 2. Core Holder Assembly

1. Load the sample into a Hassler-type core holder^41^ (Figure 1) by following the steps below.
2. Dismantle the core holder assembly by removing the sealing screw and the M4 bolts of the flowhead. Remove the sealing ring from its groove in the flowhead and clean the sealing surfaces using a clean cloth with a cleaning liquid such as acetone. Place the core holder assembly components on a clear bench in good order (see Figure 1**A** for the sealing screw, Figure 1**B** for the flowhead, Figure 1**C** for the 1/16 PEEK tubing, Figure 1**D** for the stainless steel end fitting, Figure 1**E** for the rock sample, Figure 1**F** for the rubber tubing, Figure 1**G** for the thermocouple, Figure 1**I** for the carbon fiber sleeve, and Figure 1**J** for the flexible heating jacket).
3. Wrap the flexible heating jacket around the carbon fiber sleeve.
4. Insert a thermocouple to the annulus via the base of the core holder.
5. Use a proportional-integral-derivative (PID) controller (Figure 2) that is custom built to control the temperature within ± 1 °C^21^. NOTE: Maintaining a stable temperature within ± 1 °C is important to avoid changing the interfacial tension of oil and brine that could affect the contact angle measurement^42^,^43^.
6. Thread polyether ether ketone (PEEK) tubing through the top and base of the core holder. Then, connect the PEEK tubing to the custom-made end pieces.
7. Cut a rubber tubing to a length approximately equal to the rock sample length plus the end pieces. Slide the sample gently into a rubber tubing and connect it to the end pieces. Ensure that the rubber tubing gives a tight fit over the end pieces to avoid having a leak of the confining fluid into the sample.
8. Place the thermocouple tip next to the sample to measure the temperature of the fluids within the pores.
9. Carefully assemble both ends of the core holder. Ensure that the sample is positioned at the center of the core holder to be in the scanning field of view.

### 3. Flow Apparatus and Flow Procedure

1. Prepare the flow apparatus (Figure 2) that is made up of 4 high-pressure syringe pumps (see Figure 2**A** for the oil pump, Figure 2**B** for the receiving pump, Figure 2**C** for the brine pump, and Figure 2**D** for the confining pump), a core holder assembly (see Figure 2**E**), a PID controller (see Figure 2**F**), and a CO_2_ cylinder (see Figure 2**G**), to perform waterflooding at the subsurface conditions.
2. Use a clamp to hold the core holder assembly and place it on the rotation stage inside the X-ray microtomography scanner.
3. Use the flexible PEEK tubing to connect the fluids from the pumps to the sample and the confining annulus.
4. Fill the isolated annulus gap with deionized water and vent the air out. Apply 1.5 MPa of confining pressure to squeeze the rubber tubing to prevent a flow along the sides of the core.
5. Connect the CO_2_ cylinder to the base three-way valve and flush CO_2 _at a low rate through the sample for 1 h to remove the air from the pore space.
6. Connect the brine pump (filled with 7 weight percent KI brine) to the base of the core holder via the base three-way valve and flush the air out of the brine injection line into the other side of the three-way valve before injecting the brine into the pore space. Inject the brine at 0.3 mL/min for 1 h (about 200 pore volumes) to fully saturate the sample with brine. Then, close the top and base three-way valves.
7. Pressure test the oil pump against the receiving pump to determine the equivalent pressure in both pumps before conducting any drainage (oil injection). First, connect both pumps through a two-way valve and keep the valve closed. Increase the pressure to 10 MPa in both pumps and stop the oil pump and open the two-way valve while the receiving pump is still running. Record the pressure reading of the oil pump (*i.e.*, 10.01 MPa), which is equivalent to 10 MPa in the receiving pump.
8. Establish the subsurface conditions by raising the pore pressure to 10 MPa and the temperature to 60 or 80 °C. Connect the flexible heating jacket and the thermocouple to the PID controller and apply the target value (60 or 80 °C). Connect the receiving pump (filled with KI brine) to the base three-way valve and increase the pore pressure in 1 MPa steps along with the confining pressure until achieving a pore pressure of 10 MPa and a confining pressure of 11.5 MPa. At this stage, the conditions replicate the hydrocarbon reservoir before the oil migration from the source rock.
9. Connect the oil pump to the top of the core holder via the top three-way valve and flush the oil through the other side of the valve to remove any air in the line. Increase the pressure to the tested equivalent pressure (*i.e.*, 10.01 MPa) while keeping the valve closed. Then, stop the oil pump and open the top three-way valve and start the drainage by injecting 20 pore volumes of oil using a constant flow rate of 0.015 mL/min (this rate is in the capillary-dominated flow regime) at subsurface conditions of 10 MPa and 60 or 80 °C.
10. Leave the system to reach equilibrium for at least 2 h after the oil injection and then acquire a high-resolution scan (*i.e.*, 2 µm/voxel) using an X-ray microtomography scanner. Please refer to step 4 for more details on how this is done.
11. Then, move the core holder assembly out of the X-ray microtomography scanner very carefully with all safety precautions in place, place the core holder assembly inside the oven, and reconnect the flow lines to perform the aging over 3 weeks to alter the rock wettability.
  1. To investigate the oil recovery as a function of wettability, use different aging protocols to generate different wettability conditions. Control the degree of wettability alteration (water-wet to oil-wet) by using different temperatures and oil compositions^30^,^31^,^44^.
  2. For example, to generate mixed-wet rock with more oil-wet surfaces, apply a relatively high temperature (80 °C) and inject crude oil (with a density of 830 ± 5 kg/m^3^ at 21 °C) continuously or frequently (dynamic aging) to provide a continuous supply of the polar crude oil components that can speed up the wettability alteration^45^. To generate weakly water-wet rock, use a lower temperature (60 °C) and no crude oil injection during the aging (static aging). To generate a mixed-wet reservoir rock with a mean contact angle close to 90°, perform dynamic aging with relatively heavier crude oil (with a density of 870 ± 5 kg/m^3^ at 21 °C mixed with heptane to induce asphaltene precipitation^46^,^47^,^48^) but at 60 °C^31^.
12. Once the aging process is completed, move the core holder assembly back into the X-ray microtomography scanner.
13. Conduct waterflooding at subsurface conditions. Pressure test the brine pump against the receiving pump before conducting waterflooding by following the same procedure as mentioned in step 3.7.
  1. First, connect the brine line to the base three-way valve, and connect the receiving pump to the top of the core holder via the top three-way valve.
  2. Perform waterflooding of 20 pore volumes at subsurface conditions using a constant low flow rate (*i.e.*, 0.015 mL/min), ensuring a low capillary number of approximately 10^-7^.
  3. Finally, leave the system to reach equilibrium for at least 2 h after waterflooding and acquire a high-resolution scan again at the same location. NOTE: Conducting such high-pressure and -temperature experiments requires a detailed risk assessment and rigorous testing of the whole flow apparatus outside the X-ray microtomography scanner before conducting any *in situ* experiments with all safety precautions in place.

### 4. Imaging Protocol

1. Use an X-ray microtomography scanner to acquire the three-dimensional X-ray scans at the micron scale of the reservoir rock saturated with oil and brine at subsurface conditions.
2. Find the most effective phase contrast between oil, brine, and rock by doping the brine phase, using KI to be the intermediate phase in terms of X-ray adsorption. To achieve a good contrast between oil (lowest sorption, black), brine (intermediate, dark gray) and rock (most sorbing phase, light gray), as shown in Figure 4, prepare mini-containers with a different weight percent of KI brine, and perform the scanning. The histogram of the gray-scale value should show 3 separate phases (Figure 4**b**).
  1. To prepare a contrast sample, half-fill a small cylindrical glass container (1 mL) with both oil and KI brine phases. Then, fill the other half of the container with crushed pieces of rock and mix them rigorously. Use a clean cylindrical metal to compact the mixture, avoiding any grain movement while scanning. Wear complete PPE and perform the mixing of the crude oil and the KI brine in a fume cupboard.
3. Use a relatively long carbon fiber core holder with a small diameter to allow the X-ray source to be brought as close as possible to the sample. Do not use a very long core holder, which could increase the sample movement due to rotation during the scan acquisition.
4. Use the 4X objective to acquire X-ray images at a high resolution (*i.e.*, 2 µm/voxel) sufficient to measure the effective *in situ* contact angle.Use flexible PEEK tubing as injection lines to allow a smooth 360° rotation of the core holder assembly during the scan acquisition.
5. For thin or low-density samples, use an X-ray source voltage and power of 80 kV and 7 W, respectively. For thick or high-density samples, use an X-ray source voltage and power of 140 kV and 10 W, respectively. NOTE: In this case, an X-ray source voltage of 80 kV and a power of 7 W were used.
6. To acquire the 2 µm/voxel scans, use the 4X objective with an exposure time (*i.e.*, 1.5 s or more) sufficient to obtain an X-ray radiation intensity of greater than 5,000 counts/s.
7. Use a high number of projections (at least 3,200 projections) depending on time constraints. NOTE: X-ray microtomography involves an ionizing radiation risk. Hence, an appropriate risk assessment is required to ensure a safe working environment.

### 5. Image Processing and Segmentation

1. First, reconstruct the X-ray tomography dataset using the software (**Table of Materials**) to generate three-dimensional X-ray images (.txm). Click **Browse** to import the input file (.txrm). Then, select the **Manual Center Shift** and search for the most appropriate center shift correction value to account for any sample movement during the scan acquisition.
  1. Search for the appropriate center shift value. Start with a large range (-10 to 10) and a large step size (1.0). Then narrow down the search range and the step size (0.1), until the optimal value is obtained.
  2. Reconstruct the scan using the optimal center shift value. Account for any beam hardening effects before the image reconstruction.
2. Use an appropriate segmentation method that is suitable for the specific application. To characterize the *in situ* wettability accurately, use a machine learning-based image segmentation method such TWS^32^ to turn gray-scale images to three-phase segmented images (oil, brine, and rock).Open the image in TWS - which is a Fiji (ImageJ)^32^ plugin - to segment the images without applying any noise filtering to avoid voxel averaging especially close to the three-phase contact line at which the contact angle is measured.
3. Select the random-forest algorithm and training features, such as Mean, Variance, and Edges, to apply a featured-based segmentation.
  1. Click **Settings** to find the 12 **Training Features** in the Segmentation settings (Gaussian blur, Derivatives, Structure, Difference of Gaussian, Maximum, Median, Variance, Mean, Minimum, Edges, Laplacian, and Hessian) from which to select the best training features. The selection is based on segmentation trials using different training features or a combination of them. For example, the combination of the Edges, Mean, and Variance training features was found to give the best segmentation results for this carbonate reservoir rock system.
  2. In the **Classifier options**, choose **FastRandomForest**.
  3. To add a new phase (*i.e.*, oil), click **Create new class**.
4. Label the pixels from all 3 phases (oil, brine, and rock) manually as an input to train a classifier model. Using the freehand drawing tool in ImageJ software (Fiji), highlight the 3 phases. Try to follow the shape of the phase while labeling the pixels. Once completed, click **Add to class**. Then, perform the same for the other 2 phases.
5. Apply the trained classifier to segment the whole image into 3 phases by clicking the **Train classifier** button.
6. Repeat steps 5.4 and 5.5 until good segmentation results are achieved. Click **Create result** to visualize the segmented image. Finally, click **Save as TIFF** to save the image. Look at Figure 5 to see an example of a good segmentation.
7. Make sure that the segmented images are in an 8-bit unsigned format and the 3 phases are assigned as 0, 1, and 2 for brine, rock, and oil, respectively, before measuring the *in situ* contact angle using the automated method.
  1. In the data visualization and data analysis software (**Table of Materials**), use the module **Convert Image Type** to convert the image to the **16-bit label** type. Use the **Arithmetic** module to perform the computation on the segmented image. In the **Expression**, specify the mathematical expression to change the number of the assigned phase [*i.e.*, if rock is phase 2, then a mathematical expression of 1*(a==2) means to assign rock as phase 1 instead of phase 2].
  2. Convert the three-dimensional segmented X-ray images from (.am) to binary raw un-signed data of 8-bit format (*.raw). Use the **Convert Image Type** module and, in the **Output Type**, select the option **8-bit unsigned** and click **Apply**. Export data as **Raw Data 3D** (*.raw).

### 6. Measuring the Contact Angle Distribution

1. Measure the *in situ* contact angle distribution from the segmented images using the automated contact angle method of AlRatrout* et al.*^27^ (example results are shown in Figure 6). To perform these measurements, follow the steps below, as illustrated in Figure 7.
2. Install the OpenFOAM library to perform the automatic contact angle and fluid-fluid interface curvature measurements.
3. Save the image file (*.raw) in a folder (case) which contains a header file and a folder called **System**.
  1. Open the header file and declare the number of voxels in three dimensions (*x*, *y*, and *z*), the voxel dimensions (*x*, *y*, and *z*) in microns, and the offset distance (0 0 0 for no shifting). Rename the header file as the image file.
  2. Use the folder called **System** to comply with the basic directory structure for an OpenFOAM case.
4. Make sure that there are 2 files (a *controlDict* file and a *meshingDict* file) in the system folder that contain the setting parameters. The *controlDict* file is where the run control parameters are set, including the start/end time. The *meshingDict* file is where the input and output files in each step of the algorithm are specified. Replace the file name with the new segmented image name in the *meshingDict* file for the steps explained below (Figure 7).
  1. Extract the surface (multi-zone mesh *M*) (look at Figure 7**b**).
  2. Add a **layer** near the three-phase contact line.
  3. Smooth the surface (look at Figure 7**c**).
  4. Set the required smoothing parameters that include the Gaussian radius kernel (R_Gauss_), Gaussian iterations, the Gaussian relaxation factor (β), the curvature radius kernel (R_K_), the curvature relaxation factor (γ), and curvature iterations. For more details, see AlRatrout* et al.*^27^.
5. Open a terminal from the same folder directory and type the following command, **voxelToSurfaceML && surfaceAddLayerToCL && surfaceSmoothVP**, to run the code and perform the contact angle and oil/brine curvature measurements.
  1. Look at Figure 7 to follow the computation steps of the contact angle on each vertex belonging to the contact line (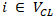) through the brine phase by: 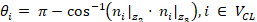 NOTE: The normal vectors are computed on the vertices comprising the contact line**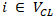. Each vertex is represented with 2 vectors normal to the oil/brine interface (*z*_2_) and the brine/rock interface (*z*_3_), as shown in Figure 7.
6. Make sure that the smooth surface file *_Layered_Smooth.vtk is generated. This file contains the measurements of the contact angle and the oil/brine interface curvature, which can be visualized using a data visualization software (**Table of Materials**), as demonstrated in Figure 7.

### 7. Quality Control

1. To be confident with the obtained automated contact angle, conduct a quality check by comparing the automated contact angle values measured from the segmented images using the AlRatrout *et al.*^27^ method to the values measured manually from raw X-ray images using the approach of Andrew* et al.*^24^.
2. To conduct the quality check, crop and segment a sub-volume from each mini-sample (Figure 8). Use the data visualization and data analysis software to crop a small sub-volume containing 1 or more oil ganglia that can be used to perform the manual contact angle measurement.
3. Run the automated code to measure the *in situ* contact angle distribution of these sub-volumes. Please refer to step 6 for how this is done.
4. Load the *_Layered_Smooth.vtk file in the data visualization software to visualize the surfaces and select the **Region** option to view the oil and brine phases, see Figure 9.
  1. Click on **Probe Location** and add the spatial coordinates (*x*, *y*, and *z*) of a randomly selected contact angle point measured using the automated contact angle method (*i.e.*, 60°). Locate its spatial location at the three-phase contact line, such as that at Figure 9**a** showing the location of the selected point (60°) as a yellow dot.
5. Then, go to the data visualization and data analysis software to conduct the manual contact angle measurement. Load the segmented sub-volume image.
6. Filter the noise from the raw X-ray image using a noise reduction filter to be used for the manual contact angle measurement only. NOTE: A non-local means filter^49^,^50^ was applied in this case.
7. Use the segmented image to render the rock transparent and only visualize the oil and brine phases to help in identifying the location of the selected point, as shown in Figure 9**b**.
  1. Use the **Arithmetic** module to perform the calculation on the segmented image. In the **Expression**, specify the mathematical expression to isolate the oil and brine phases separately [*i.e.,* the mathematical expression **a==1** means isolate phase 1 (brine in this case)].
  2. Then, use the module **Generate Surface** to generate the oil and brine surfaces, and use the module **Surface View** to visualize the oil and brine surfaces in the desired colors.
8. Once the location of the point is identified, bring the filtered-raw X-ray image slice to the same location, as shown in Figure 9**c**.
  1. Open the module **Slice** and change the **Translate** value.
9. Extract the three-phase contact line using the **Label Interfaces** module on the segmented image.
  1. Type **3** in the **Number of Phases** box. Select **No** in the **Only Black Voxels**, apply and open the **Isosurface** module on the labeled interfaces, and change the **Colormap** and **Threshold** values as desired for the effective visualization.
10. In the **Slice** module, turn on the **Plane Definition**, and in the options, select **Show dragger**. Hold the dragger and move it to the desired location at which that manual contact angle will be measured.
  1. In the **Display Options**, select the rotate option. Hold the rotate handle to rotate the slice.Rotate the slice to be perpendicular to the three-phase contact line and measure the contact angle manually using the angle measurement tool as shown in Figure 9**d**. NOTE: Here, the contact angle was found to be 61°.
11. Plot the manually measured contact angle against the automated contact angle value measured at the same location to confirm the accuracy of the automated contact angle measurements. Look at Figure 10 to observe the comparison measurements of the contact angle between the automated method and the manual method of the sub-volume from mini-sample 1.

## Representative Results

For the 3 samples studied, the measured *in situ* distribution of the contact angle is shown in Figure 6, with the oil recovery shown in Figure 11. Figure 12 shows images of the remaining oil distributions for different wetting conditions at the end of the waterflooding. The mixed-wettability (or the range of the contact angle) was measured using the automated contact angle method^27^. The measured contact angle distributions are considered to be representative results if there is a good match between the contact angle points measured using the automated method from segmented images compared to the manually measured contact angles from raw X-ray images. Figure 10 shows an example of a good match of a comparison measurement between the automated contact angles and the manual contact angles at the same locations for a sub-volume from mini-sample 1 (weakly water-wet).

Three aging protocols were performed to treat the 3 samples and generate 3 wetting conditions (Figure 6). Aging the sample at a lower temperature (60 °C) and statically (no oil injection during the aging period) could result in a weakly water-wet condition, such as the distribution shown for sample 1 in blue (Figure 6). On the other hand, aging the sample at a higher temperature (80 °C) and with partially dynamic aging (an oil injection during the aging period) could result in mixed-wet conditions with more oil-wet surfaces, like that of sample 2 shown in gray (Figure 6).

The oil recovery was found to be a function of wettability, similar to earlier core-scale studies^51^. However, at that time, the oil recovery was shown as a function of the core-scale wettability index. Similar oil recovery behavior has been observed at the pore scale and was plotted as a function of the mean value of the *in situ* contact angle distribution (Figure 11). The low oil recovery of sample 1 (weakly water-wet) was due to the trapping of oil in larger pore spaces. The brine percolated through the small pore corners, leaving the oil trapped as disconnected ganglia in the center of the pore spaces with quasi-spherical shapes (Figure 12**a**), similar to what has been observed in previous investigations in water-wet media^52^,^53^,^54^,^55^. In contrast, sample 2 (a mixed-wet case with more oil-wet surfaces) had oil layers that were largely connected (Figure 12**b**). These thin layers only allowed a slow oil production, leaving a high remaining oil saturation at the end of the waterflooding. The highest oil recovery was achieved in sample 3 (mixed-wet with a mean contact angle close to 90°) which was neither water-wet (so there is less trapping in large pores) nor strongly oil-wet (less oil is retained in small pore spaces)^1^. In the mixed-wet cases of sample 2 and 3, oil was left in connected, thin sheet-like structures (Figure 12b and **12c**) similar to other studies in oil-wet porous media^52^,^53^,^56^.

**Figure:**
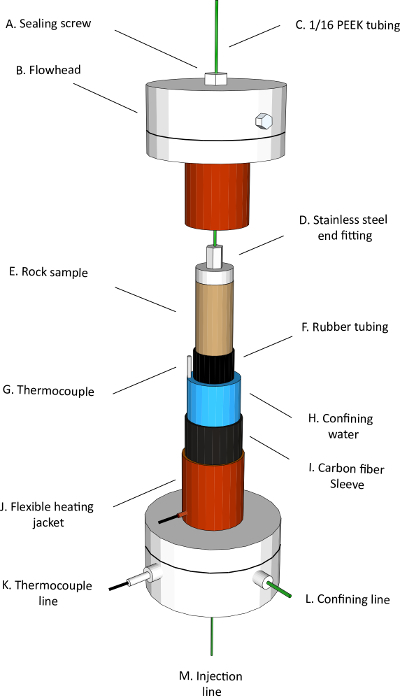

Figure 1**: A schematic illustration diagram of the core holder assembly.** Components of the core holder are labeled, and the internal cross-section view of the core holder is shown. Please click here to view a larger version of this figure.

**Figure:**
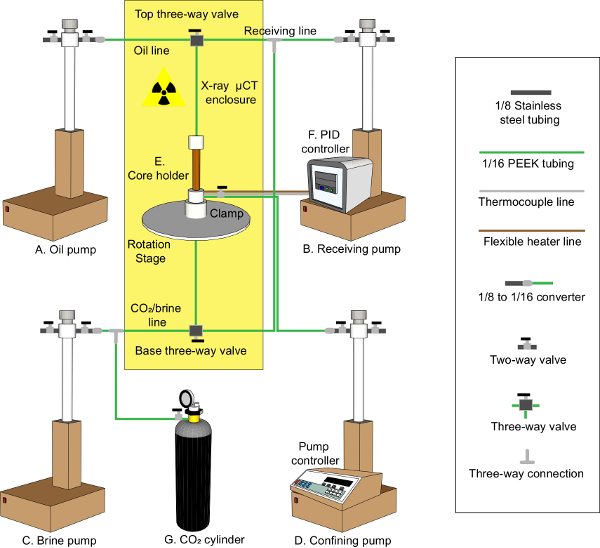

Figure 2**: The high-pressure, high-temperature flow apparatus.** The flow apparatus is comprised of four high-pressure syringe pumps: (**A**) an oil pump, (**B**) a receiving pump, (**C**) a brine pump, and (**D**) a confining pump. Panel (**E**) shows the core holder assembly, (**F**) shows the PID controller, and (**G**) shows the CO_2_ cylinder. Please click here to view a larger version of this figure.

**Figure:**
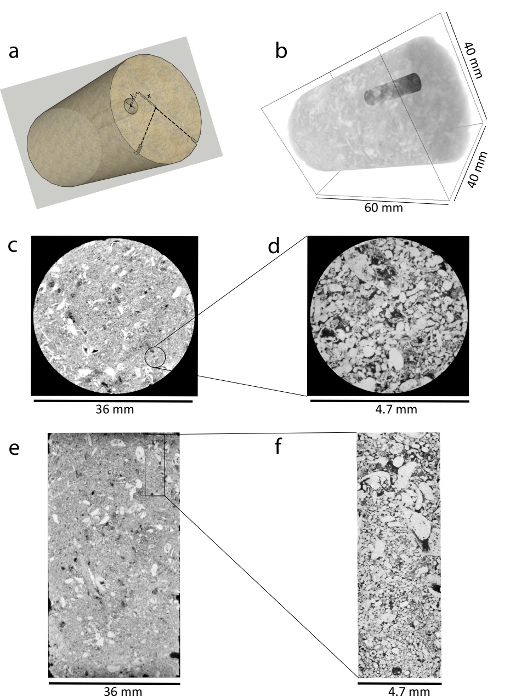

Figure 3**: Images demonstrating the drilling of representative mini-samples.** (**a**) This cartoon illustrates the orthogonal marks with a good drilling location. *x* and *y* are the distances from the center of the core plug used to find where to drill. (**b**) This panel shows a dry X-ray three-dimensional image of the core plug (rendered semi-transparent) with a mini-sample (in dark gray). (**c**) This is a horizontal cross-sectional view of the core plug (scanned at 40 µm/voxel). The rock grains and pores are shown in gray and black, respectively. (**d**) This panel shows a horizontal cross-sectional view of the mini-sample (scanned at 5.5 µm/voxel). (**e**) This is a vertical cross-sectional view of the core plug showing the complex and heterogeneous pore sizes and geometries along with the location of the mini-sample indicated by the black box. (**f**) This is a magnified vertical cross-sectional view of the highlighted mini-sample shown in panel **e** that was scanned at 5.5 µm/voxel. Please click here to view a larger version of this figure.

**Figure:**
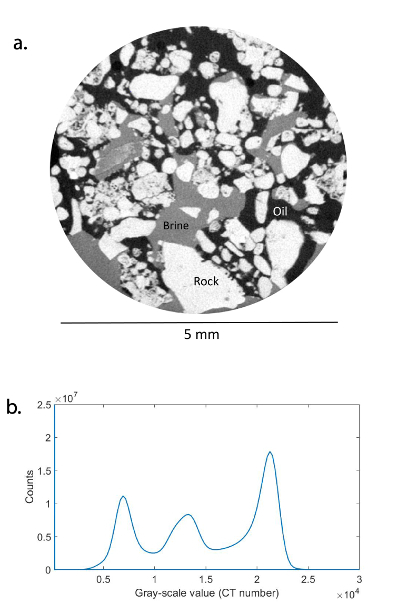

Figure 4**: A phase contrast scan.** (**a**) This panel shows a contrast scan of crushed rock (light gray) mixed with brine (dark gray) and oil (black) phases. This was used to determine the appropriate doping of the brine to ensure a good phase contrast. (**b**) This is a histogram of the gray-scale value of the three phases. Please click here to view a larger version of this figure.

**Figure:**
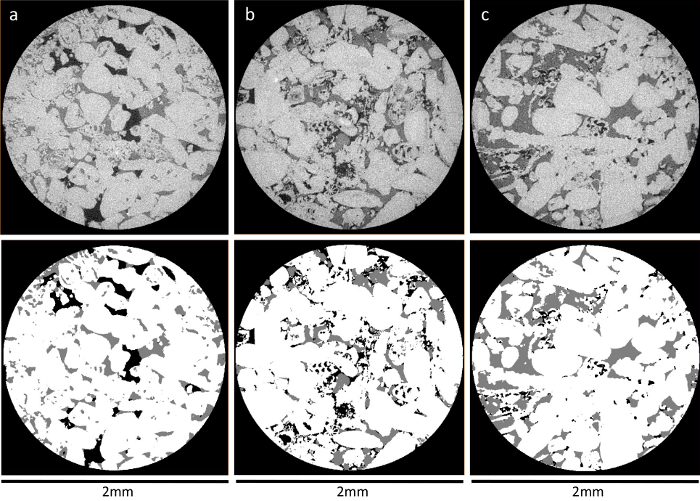

Figure 5**: A horizontal cross-sectional view of raw and segmented X-ray images of three mini-samples.** Panels (**a**), (**b**), and (**c**) show *xy* cross-sectional views of mini-samples 1, 2, and 3, respectively. The top row shows the raw gray-scale X-ray images (oil, brine, and rock, are in black, dark gray, and light gray, respectively). The lower images show the segmented images of the same slice using Trainable WEKA Segmentation (oil, brine, and rock, are in black, gray, and white, respectively). Please click here to view a larger version of this figure.

**Figure:**
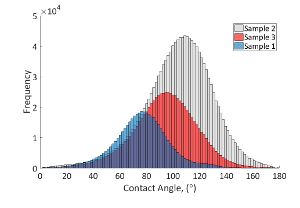

Figure 6**: Distributions of the contact angle measurement of the three mini-samples. **Sample 1 has a mean contact angle of 77° ± 21° with 462,000 values shown in blue. Sample 2 has a mean contact angle of 104° ± 26° with 1.41 million values shown in gray. Sample 3 has a mean contact angle of 94° ± 24° with 769,000 values shown in red. Please click here to view a larger version of this figure.

**Figure:**
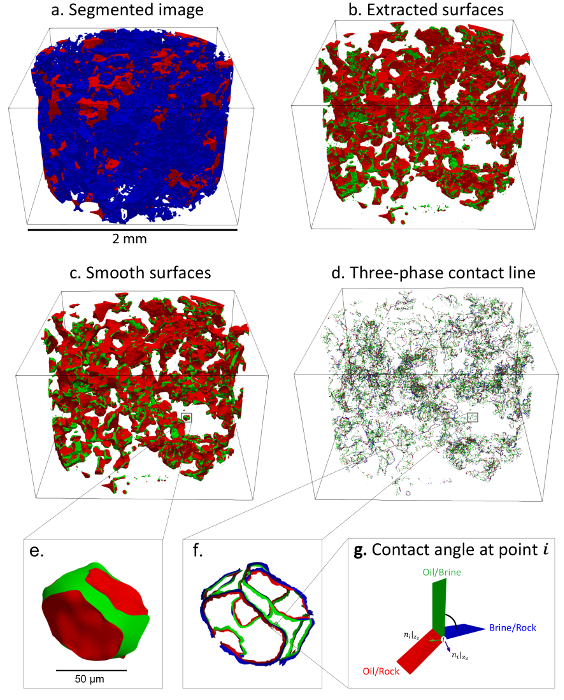

Figure 7**: The workflow for an automated contact angle measurement.** (**a**) This is a three-dimensional segmented image showing brine in blue and oil in red, while rock is rendered transparent. (**b**) This panel shows extracted surfaces of the whole image. The oil/brine surfaces are shown in green, while the oil/rock surfaces are shown in red. (**c**) This panel shows the smoothed surfaces of the whole image. (**d**) This panel shows the three-phase contact line of the whole image. (**e**) This is an example of the smoothed surfaces of an oil ganglion highlighted by the black square. (**f**) This panel shows the three-phase contact line of the highlighted oil ganglion. (**g**) This is an example of a single contact angle measuring at point *i* (highlighted in panel **f**)**. **The oil/brine, oil/rock, and brine/rock surfaces are shown in green, red, and blue, respectively. Please click here to view a larger version of this figure.

**Figure:**
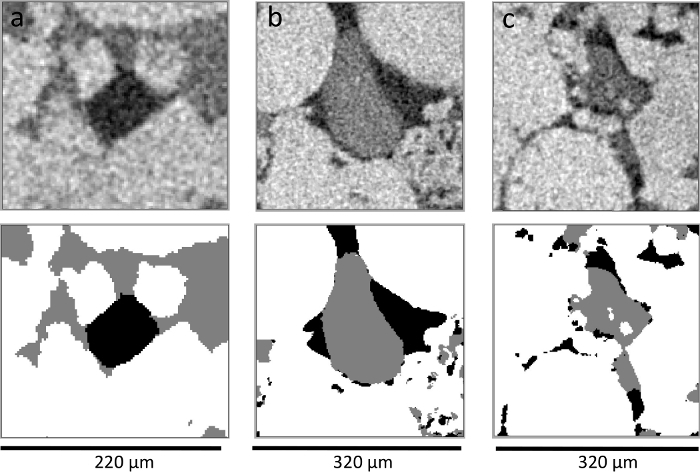

Figure 8**: Three sub-volumes extracted from the three mini-samples.** (**a**) This panel shows the sub-volume extracted from mini-sample 1 (weakly water-wet). (**b**) This panel shows the sub-volume extracted from mini-sample 2 (mixed-wet). (**c**) This panel shows the sub-volume extracted from mini-sample 3 (mixed-wet)*.* Please click here to view a larger version of this figure.

**Figure:**
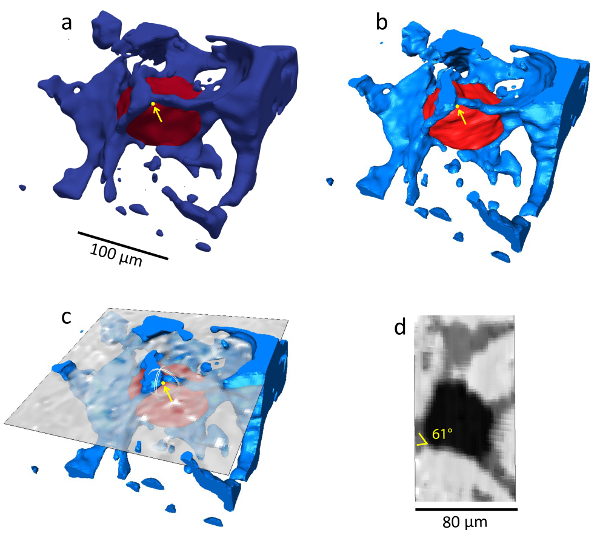

Figure 9**: A one-to-one contact angle measurement workflow.** (**a**) This is a visualization of a randomly selected contact angle point (60°) measured using the automated code (the image is obtained from the data visualization software used). (**b**) This panel shows how to identify the location of the same point using the data visualization and analysis software. (**c**) This panel shows how to conduct a manual contact angle measurement at the same location. (**d**) This is an example of the manually measured contact angle point at the same location (61°). Please click here to view a larger version of this figure.

**Figure:**
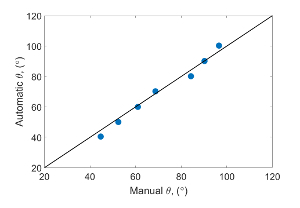

Figure 10**: Automated contact angle measurements compared to the manual contact angle measurements at the same locations of the sub-volume from mini-sample 1.** The values were measured following the procedure described in Figure 9. Please click here to view a larger version of this figure.

**Figure:**
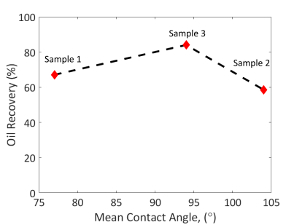

Figure 11**: Oil recovery as a function of wettability.** The oil recoveries of sample 1, 2, and 3 are 67.1%, 58.6%, and 84.0%, respectively. Please click here to view a larger version of this figure.

**Figure:**
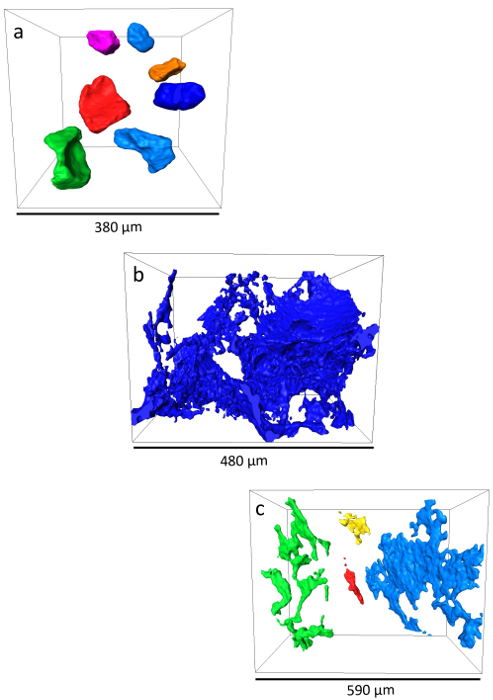

Figure 12**: The remaining oil morphology for different wetting conditions.** (**a**) In sample 1 (weakly water-wet), the remaining oil was trapped at the center of the pores as disconnected ganglia with quasi-spherical shapes. Panels (**b**) and (**c**) show how in samples 2 and 3 (mixed-wet), the remaining oil was left in connected, thin sheet-like structures in small pores and crevices. The different colors represent disconnected oil ganglia. Please click here to view a larger version of this figure.

## Discussion

The most critical steps for an *in situ* wettability characterization at high pressure and temperature to be successful are as follows. 1) Generate a good image segmentation that is essential to obtain accurate contact angle measurements. 2) Avoid including large impermeable grains in the mini-samples that could seal off the flow, and large vugs resulting in a very fragile sample with non-representative porosity. 3) A well-controlled flow experiment with no leaks is important because mini-samples are very sensitive to the amount of injected fluid (*i.e.*, one pore volume is about 0.1 mL). 4) Avoid the presence of air (as a fourth phase) in the pore space. 5) Maintain a temperature control of the sample during the whole flow experiment. 6) Avoid any interface relaxation during the scan acquisition by waiting for the system to reach equilibrium. 7) Use an appropriate center shift correction, which is necessary for the effective X-ray image reconstruction.

The automated contact angle method is limited by the accuracy of the image segmentation because it is applied to segmented images only. Image segmentation depends largely on imaging quality that depends on the imaging protocol and the performance of the microtomography scanner. Furthermore, it is sensitive to the image reconstruction and the noise reduction filters, as well as the segmentation method such as the TWS^32^ or the seeded watershed method^57^. In this work, the TWS method provided more accurate contact angle measurements on raw X-ray images compared to those by a watershed method applied to filtered X-ray images (using noise reduction filters). The use of noise reduction filters makes the interface appear to be less oil-wet at some parts of the rock, due to the voxel averaging especially close to the three-phase contact line^31^. TWS can capture not only the amount of remaining oil saturation but also the shape of the remaining oil ganglia. This is especially the case for the remaining oil in the mixed-wet cases, in which oil is retained in the pore space as thin sheet-like structures, making it a challenge to be segmented based on gray-scale threshold values only.

This *in situ* wettability determination provides a thorough description of the wetting conditions of reservoir rocks compared to other conventional wettability measurement methods. It takes into account all important pore-scale rock parameters, such as rock surface roughness, rock chemical compositions, and pore size and geometry, that are not possible by wettability indices^7^,^8^ and *ex situ* contact angle methods^4^,^9^,^10^,^11^. The use of an automated *in situ* contact angle measurement at the micron scale is robust and removes any subjectivity associated with the manual method^24^. Moreover, it is more effective in removing voxelization artefacts compared to other automated methods^25^,^26^. The *in situ* contact angle distribution measured using the automated method was relatively rapid. For example, the runtime for measuring the contact angle on any of the three sample images that contain 595 million voxels is approximately 2 h, using a single 2.2 GHz CPU processor.

In the future, this protocol can be used to characterize other reservoir rock systems saturated with formation brine and crude oil. The same method is not limited to the petroleum industry only and can be modified and adapted to characterize the wettability from any segmented three-dimensional images with two immiscible fluids in porous media with a variety of wettability conditions.

## Disclosures

The high resolution X-ray micro-tomography datasets reported in this paper are available at the Digital Rocks Portal: www.digitalrocksportal.org/projects/151  The codes used to run automatic measurements of contact angle and fluid/fluid interface curvature are available at GitHub: https://github.com/AhmedAlratrout/ContactAngle-Curvature-Roughness 
